# High-Flow Nasal Oxygen in Contemporary Airway Management: Enhancing Physiological Safety in Anaesthesia—A Narrative Review

**DOI:** 10.3390/healthcare14142206

**Published:** 2026-07-21

**Authors:** William P. L. Bradley, Jane Anderson, Maryanne Balkin, David J. Brewster, Eugenie Kayak, Ina I. Shariffuddin, Patrick C. F. Tan, Patrick Wong

**Affiliations:** 1 Department of Anaesthesiology and Perioperative Medicine, The Alfred, Melbourne, VIC 3004, Australia; 2 School of Translational Medicine, Faculty of Medicine Nursing and Allied Health, Monash University, Melbourne, VIC 3004, Australia; 3 Department of Anaesthesia and Pain Medicine, Epworth Healthcare, Melbourne, VIC 3121, Australia; 4 Department of Critical Care, The University of Melbourne, Melbourne, VIC 3010, Australia; 5 Intensive Care Unit, Cabrini Hospital, Melbourne, VIC 3144, Australia; 6 Department of Anaesthesia, Austin Health, Melbourne, VIC 3084, Australia; 7 Department of Anaesthesiology, Faculty of Medicine, Universiti Malaya, Kuala Lumpur 50603, Malaysia; 8 Medical Education & Research Development Unit, Faculty of Medicine, Universiti Malaya, Kuala Lumpur 50603, Malaysia; 9 Department of Anaesthesia, The Royal Women’s Hospital, Melbourne, VIC 3052, Australia; 10 Department of Anaesthesia and Pain Medicine, Waikato Hospital, Hamilton 3204, New Zealand

**Keywords:** high-flow nasal oxygen, apnoeic oxygenation, respiratory physiology

## Abstract

High-flow nasal oxygen (HFNO) has become an increasingly important component of contemporary airway management, extending beyond its origins as a therapy for hypoxaemic respiratory failure to a versatile perioperative tool used across operating theatres, non-operating-room anaesthesia (NORA), and critical care settings. By delivering heated, humidified oxygen at high flow rates, HFNO provides a range of physiological benefits including reliable oxygen delivery, anatomical dead-space washout, low-level positive airway pressure, and facilitation of apnoeic oxygenation. This narrative review explores the physiological mechanisms underpinning HFNO and examines its evolving role in modern anaesthetic practice. The evidence supporting its use for preoxygenation, apnoeic oxygenation, postoperative respiratory support, procedural sedation, tubeless airway surgery, and management of the physiologically difficult airway is reviewed. Particular attention is given to high-risk populations, including patients living with obesity, obstetric patients, and children, as well as applications in a range of NORA environments. The review also examines key limitations and safety considerations, including hypercapnia, aspiration risk, airway obstruction, airway-fire hazards, human factors, and emerging concerns regarding environmental sustainability. While HFNO can improve oxygenation and increase the margin of safety during periods of physiological vulnerability, it should not be viewed as a substitute for definitive airway management or vigilant monitoring. HFNO represents a significant advance in physiology-centred airway management. Its greatest value lies not in routine application, but in thoughtful integration into broader airway safety strategies where meaningful physiological or patient-centred benefit is anticipated.

## 1. Introduction

High-flow nasal oxygen (HFNO) has become an integral component of modern airway management in anaesthesia and critical care [1,2,3]. Originally developed as a therapy for hypoxaemic respiratory failure, it has rapidly transitioned into a perioperative tool that enhances oxygenation during periods of physiological vulnerability [4]. Its adoption has expanded across operating rooms (ORs) and non-operating-room anaesthesia (NORA) settings, particularly for procedural sedation.

HFNO delivers heated, humidified oxygen at high flow rates, typically up to 60–70 L min^−1^ in adults, via wide-bore nasal cannulae. Unlike conventional low-flow systems, it provides a stable and predictable inspired oxygen fraction (FiO_2_), even in patients with high inspiratory flow demand. Active humidification improves tolerance and preserves mucosal function, enabling sustained delivery in both spontaneously breathing and apnoeic patients.

The clinical value of HFNO lies in its physiological effects. High flow rates reduce nasopharyngeal dead space, improve alveolar oxygen delivery, and generate a modest degree of flow-dependent positive airway pressure [5,6], which may increase functional residual capacity (FRC). During apnoea, continuous oxygen insufflation supports ongoing diffusion into the alveoli, prolonging safe apnoea time. Together, these mechanisms increase the margin of safety during airway manipulation and sedation.

In contemporary practice, HFNO has been particularly influential in four key areas:

1. Preoxygenation before tracheal intubation;
2. Apnoeic oxygenation during laryngoscopy and airway instrumentation;
3. Post-extubation and post-procedural respiratory support;
4. Procedural sedation, especially in NORA settings.

Traditional facemask preoxygenation requires interrupting oxygen delivery at the onset of laryngoscopy. HFNO allows oxygen administration to continue throughout airway instrumentation, extending the duration before desaturation and improving safety in patients at risk of rapid hypoxaemia.

The concept of the physiologically difficult airway recognises that hypoxaemia, reduced FRC, obesity, pregnancy, or increased metabolic demand may make even technically straightforward intubation hazardous. In these patients, time to desaturation, not anatomical difficulty, determines outcome. HFNO directly addresses this risk by improving oxygen reserves and prolonging safe apnoea.

Hence, the expanding role of HFNO in procedural sedation and general anaesthesia reflects a broader transition from conventional oxygen therapy (COT) toward physiology-centred airway management to extend safe apnoea time, mitigate hypoxaemia, and improve perioperative airway safety.

Importantly, HFNO is not a substitute for careful airway planning. It does not prevent carbon dioxide accumulation, nor does it replace optimal positioning or rescue preparedness. Rather, its strength lies in integration within a system-wide airway safety strategy.

As its use continues to expand, understanding the physiological principles, evidence base, and limitations of HFNO is essential for safe and effective clinical application.

## 2. Methodology

This article was developed as a narrative review and expert synthesis of the role of HFNO in contemporary airway management, with a particular focus on anaesthesia, perioperative care, procedural sedation, and non-operating-room anaesthesia environments. The aim was to provide a clinically focused summary of the physiological rationale, current evidence base, practical applications, safety considerations, and environmental implications of HFNO use.

A targeted literature search was undertaken using PubMed/MEDLINE, Google Scholar, and relevant professional society guidelines. The search focused primarily on literature published within the last 10 years, while also including earlier relevant 21st-century publications that informed the physiological rationale, mechanisms of action, foundational evidence, or the evolution of HFNO practice. Search terms included combinations of “high-flow nasal oxygen”, “high-flow nasal cannula”, “HFNO”, “HFNC”, “THRIVE”, “apnoeic oxygenation”, “preoxygenation”, “perioperative oxygenation”, “procedural sedation”, “non-operating room anaesthesia”, “NORA”, “difficult airway”, “physiologically difficult airway”, “obesity”, “pregnancy”, “paediatrics”, “extubation”, “airway fire”, “environmental impact”, and “sustainability”. Additional references were identified through citation tracking of key articles, guidelines, and review papers.

Articles were selected based on clinical relevance, methodological quality, and applicability to contemporary anaesthetic practice. Priority was given to publications addressing the physiological mechanisms of HFNO, clinical outcomes in anaesthetic or perioperative settings, use in high-risk patient groups, procedural sedation, airway safety, implementation in NORA environments, medico-legal implications, and environmental sustainability. Relevant randomised trials, observational studies, systematic reviews, meta-analyses, narrative reviews, society guidelines, professional guidance documents, and expert consensus statements were considered.

Given the breadth and heterogeneity of HFNO applications, a formal systematic review or meta-analysis was not undertaken. Instead, the evidence was synthesised narratively, with emphasis on areas of consistent clinical benefit, areas of uncertainty, and situations in which HFNO may add physiological safety or procedural value. Particular attention was given to the distinction between HFNO as an adjunct to airway management and its limitations as a substitute for adequate monitoring, airway assessment, or definitive airway control.

The review also incorporated expert opinion from the author group, based on available evidence, clinical experience, and relevant national professional guidance. The author group included contributors with experience in developing ANZCA guidance on airway management and fire safety, and clinicians recognised as being at the forefront of their respective fields in airway management, physiological difficult airway practice, obstetric HFNO, anaesthesia and the law, environmental sustainability in healthcare, and paediatric anaesthesia. This multidisciplinary perspective informed the interpretation of the literature and the synthesis of practical recommendations, particularly in areas where evidence is evolving or where practice is guided by physiology, safety principles, and expert consensus.

## 3. Physiological Mechanisms

**Figure 1:**
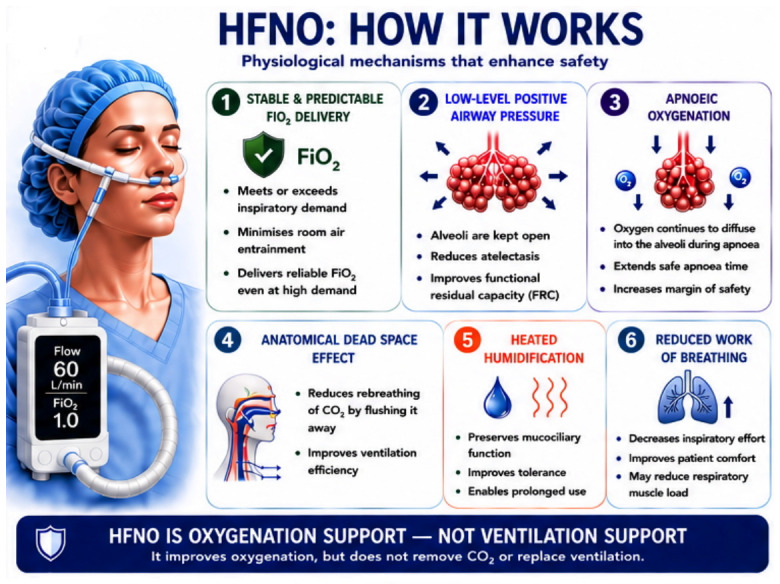
HFNO physiological mechanisms.

HFNO exerts its clinical effects through multiple overlapping and synergistic physiological mechanisms. Rather than functioning solely as a high-concentration oxygen delivery device, HFNO influences oxygenation, ventilation, lung volumes, respiratory mechanics, and airway conditioning. The principal mechanisms include accurate and stable FiO_2_ delivery [7], anatomical dead-space washout [8], generation of low-level positive airway pressure [5,6], preservation of mucociliary function through heated humidification [9,10], effects on FRC, facilitation of apnoeic oxygenation, reduction in work of breathing [11], and important limitations in carbon dioxide clearance (Figure 1).

One of the primary advantages of HFNO is its ability to deliver a reliable and predictable fraction of inspired oxygen. In conventional low-flow oxygen systems, inspiratory demand often exceeds the delivered flow, leading to ambient air entrainment and variability in the effective FiO_2_. This inconsistency becomes particularly problematic in tachypnoeic or hypoxaemic patients, whose inspiratory flow rates are high. HFNO mitigates this limitation by delivering flow rates that meet or exceed peak inspiratory demand, thereby minimising room air entrainment. As a result, the delivered FiO_2_ more closely approximates the set FiO_2_, and oxygen concentration within the nasopharynx remains stable throughout the respiratory cycle. This reliability is especially important in acute hypoxaemic respiratory failure, peri-intubation optimisation, and procedural sedation, where preservation of oxygen reserve is critical.

An additional key mechanism is anatomical dead-space washout. The nasopharynx and oropharynx comprise a substantial portion of anatomical dead space, and during normal respiration, exhaled carbon dioxide-rich gas remains within this region and is partially rebreathed during the next inspiration. HFNO delivers continuous high-flow during expiration, effectively flushing carbon dioxide-rich gas from the upper airway, reducing rebreathing, and increasing the oxygen concentration available for the subsequent inspiratory effort. This improves ventilatory efficiency and may contribute to modest reductions in PaCO_2_ in spontaneously breathing patients. Dead-space washout is particularly relevant in high-minute-ventilation states and during sedation-related hypoventilation.

Although HFNO is an open system, it generates measurable positive airway pressure. The magnitude of this pressure depends on flow rate [6], mouth position (higher pressures are observed when the mouth is closed), cannula fit, and upper airway compliance. As a general approximation, positive airway pressure increases by around 0.7–1 cm H_2_O for every 10 L min^−1^ increase in flow [6], although this relationship varies and is patient-dependent. This low-level positive airway pressure increases end-expiratory lung volume, partially recruits alveoli, reduces atelectasis formation, and improves ventilation–perfusion matching. These effects can increase FRC, which serves as the primary oxygen reservoir during apnoea. Even modest increases in FRC may meaningfully prolong safe apnoea time, particularly in obese or obstetric patients where baseline lung volumes are reduced. However, HFNO should not be equated with continuous positive airway pressure (CPAP) or non-invasive ventilation, as it does not provide controlled, sustained positive airway pressure or inspiratory pressure support.

HFNO also facilitates apnoeic oxygenation through the principle of mass flow. During apnoea, oxygen continues to diffuse from the alveoli into pulmonary capillary blood at a rate of approximately 200–250 mL min^−1^, while net carbon dioxide transfer into the alveoli is significantly lower due to buffering in blood and tissues. This imbalance creates a slight subatmospheric gradient within the alveoli, drawing oxygen from the pharynx into the lungs even in the absence of diaphragmatic movement. By maintaining a continuous reservoir of high FiO_2_ within the upper airway, HFNO enhances this process, allowing oxygen delivery to continue during laryngoscopy and intubation attempts. However, it does not eliminate carbon dioxide, and progressive hypercapnia and respiratory acidosis will develop during prolonged apnoea.

Heated humidification is fundamental to both physiological efficacy and patient tolerance [12,13]. HFNO systems deliver gas heated to approximately 37 °C and fully humidified to near-physiological absolute humidity. This is essential because cold, dry gas impairs mucociliary clearance, promotes epithelial injury, increases airway resistance, causes secretory retention, and reduces patient comfort. In contrast, heated humidification preserves epithelial integrity, maintains ciliary function, improves secretion mobilisation, and enhances tolerance of high flows. Maintenance of mucosal function may support airway defence mechanisms and improve tolerability during prolonged HFNO use in acute and perioperative settings.

HFNO reduces the work of breathing by matching inspiratory flow demand, decreasing effective dead space, and providing low-level positive airway pressure. These combined effects may lower the respiratory rate, reduce accessory muscle use, and improve comfort in spontaneously breathing patients.

Despite these benefits, HFNO has clear limitations. It does not provide active ventilation or inspiratory pressure support and cannot compensate for severe hypoventilation, complete airway obstruction, or established ventilatory failure. Although modest reductions in PaCO_2_ may occur in selected patients through improved ventilatory efficiency, HFNO does not prevent progressive carbon dioxide accumulation during true apnoea. In hypercapnic respiratory failure, non-invasive ventilation remains the preferred modality where clinically appropriate.

Although HFNO is often discussed alongside COT and non-invasive ventilation, these modalities differ substantially in their mechanisms, clinical roles and limitations. Table 1 summarises the key practical distinctions relevant to anaesthetic and procedural practice. Accordingly, HFNO should be viewed as an oxygenation strategy rather than a substitute for ventilatory support, airway protection, or definitive airway management when these are clinically indicated.

## 4. Advances in Equipment and Integration into Anaesthetic Workflow

Technological refinement has been central to the expansion of HFNO into perioperative and procedural practice. Developments in flow delivery, oxygen blending, humidification systems, circuit design, and interface engineering have improved physiological performance while allowing safer integration into anaesthetic workflows.

### 4.1. Flow Systems and FiO_2_ Delivery

Modern HFNO systems utilise integrated air–oxygen blenders to deliver precise, adjustable FiO_2_ across a wide range of flow rates, typically 60–80 L min^−1^. Accurate blending ensures that the set FiO_2_ more closely reflects the delivered FiO_2_, even during periods of high inspiratory demand, such as anaesthesia induction or respiratory distress. Rapid titration of both flow and oxygen concentration enables dynamic adjustment during preoxygenation, airway manipulation, and emergence. Reliable high-flow generation remains fundamental to minimising air entrainment and maintaining a stable oxygen reservoir within the nasopharynx.

### 4.2. Heated Humidification

Contemporary humidification systems actively heat and saturate the delivered gas to near-physiological temperatures and humidity levels. This preserves mucociliary function, reduces airway irritation, and permits sustained delivery of high flows with improved patient tolerance [14]. Improved thermal control mechanisms enhance consistency across varying ambient OR conditions and reduce fluctuations in gas conditioning.

### 4.3. Circuit Considerations

Circuit design has evolved to improve thermal stability and minimise condensation. Heated and insulated tubing reduces gas cooling below its dew point, thereby limiting condensation and maintaining consistent humidity delivery.

Interface design has also advanced. Compressible nasal cannula segments and integrated flow-diverter mechanisms enable the use of a facemask without removing the prongs, facilitating rapid escalation to facemask ventilation [15]. By diverting high-flow oxygen away from the closed mask circuit, these systems reduce unnecessary circuit pressurisation and mitigate the theoretical risk of gastric insufflation associated with uncontrolled high flow within a sealed system.

Asymmetrical nasal cannulae, incorporating different-sized prongs, are designed to optimise intranasal flow dynamics. By modifying how gas distributes within the nasopharynx, these designs may enhance dead space washout and modestly increase expiratory resistance, potentially augmenting the effects of low-level positive airway pressure [16]. Appropriate sizing remains important, with prongs typically occupying no more than approximately half of the nares diameter to permit adequate gas egress and avoid excessive pressure generation.

### 4.4. Or Integration

HFNO is increasingly incorporated into routine anaesthetic practice. It can be maintained during preoxygenation, laryngoscopy, and early airway management, reducing interruption in oxygen delivery. The ability to transition seamlessly to facemask ventilation without removing the interface enhances safety during induction and airway rescue, particularly in high-risk patients. Compact design and compatibility with existing anaesthetic infrastructure have further supported its integration into both OR and NORA environments.

## 5. Safety, Contraindications and Limitations

**Figure 2:**
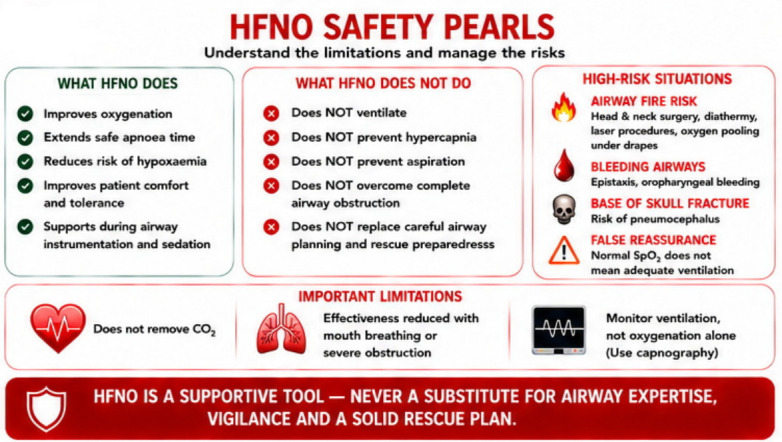
HFNO limitations.

Several relative contraindications and situational limitations should be considered when selecting HFNO (Figure 2).

HFNO relies on at least a partially patent upper airway to be effective. Its mechanisms, high-flow oxygen delivery, dead-space washout, and generation of low-level positive airway pressure depend on gas flow to the lower airway. In the presence of significant dynamic or fixed obstruction, oxygen delivery may be markedly reduced. It should not be viewed as a solution to mechanical airway compromise [17], but rather as a technique to buy time while preparations are made for definitive airway management. Clinicians must therefore remain prepared to escalate promptly if airway patency cannot be maintained.

Bleeding within the upper airway presents important practical and safety concerns. Active epistaxis or oropharyngeal bleeding may be exacerbated by high gas flows, which can aerosolise blood. Aerosolised blood poses both an infection-control risk and an occupational hazard to OR staff. In addition, insertion of adjuncts such as nasopharyngeal airways to improve patency carries a recognised risk of mucosal trauma and further bleeding, particularly in anticoagulated patients or those with friable mucosa. In such settings, a more controlled and protected airway approach is preferable.

Severe facial trauma with risk of base of skull fractures warrants particular caution in addition to bleeding considerations. Although HFNO generates relatively low levels of positive airway pressure compared with non-invasive ventilation, there remains a risk of intracranial air entry (pneumocephalus) if the skull base is disrupted, which has occurred in both neonates and adults [18,19,20]. While rare, this possibility supports avoiding HFNO in patients with suspected or confirmed skull base injury.

Airway protection and aspiration risk must also be explicitly considered. HFNO provides oxygenation but does not protect against aspiration or increase the risk of gastric insufflation [21,22,23,24]. In patients at increased risk of regurgitation, such as those with delayed gastric emptying, bowel obstruction, morbid obesity, or recent oral intake, sedation combined with HFNO may maintain oxygen saturation while failing to mitigate aspiration risk. This consideration has become increasingly relevant with the widespread use of glucagon-like peptide-1 (GLP-1) receptor agonists [25,26], which are associated with delayed gastric emptying. In selected patients, a secured airway with a cuffed endotracheal tube may be a safer strategy than procedural sedation with high-flow oxygen alone [26].

Airway fire risk is an additional, critical consideration [27,28,29,30]. HFNO delivers high concentrations of oxygen directly to the upper airway, creating an oxygen-enriched environment around the patient’s face, chest, and surgical field [31]. During procedures involving electrocautery, diathermy, or laser, particularly in the head, neck, and upper chest regions, the fire triad of oxidiser, ignition source, and fuel will be present [32].

This risk is also relevant during procedural sedation when high-flow oxygen is administered under drapes or in close proximity to the operative site [33]. Even when the procedure does not directly involve the airway, oxygen pooling beneath surgical drapes can significantly increase local oxygen concentration and increase the risk of combustion. Safe practice requires the use of the lowest effective FiO_2_, careful draping that allows gas egress, explicit communication with the proceduralist before activation of ignition sources, and readiness to immediately reduce or discontinue oxygen delivery if required. Structured shared-airway and fire-safety protocols are strongly recommended [29,34,35]. To support clinical implementation, a Practical Precautions Summary for HFNO use during airway-adjacent, head and neck, facial, dental, and upper chest procedures is provided in Appendix A.

Importantly, the need for supplemental oxygen during procedural sedation in the head, neck and upper chest region should be actively reconsidered once adequate local anaesthesia has been established. If the patient is maintaining airway patency and effective spontaneous ventilation, additional oxygen may not be necessary. Continuing high-flow oxygen in the presence of ignition sources unnecessarily increases the risk of fire; therefore, oxygen should be used only when clinically indicated and at the lowest effective FiO_2_. If supplemental oxygen is anticipated during a procedure with a high fire risk, it may be safer to secure the airway from the outset with a supraglottic airway device or a cuffed endotracheal tube [29,35], allowing controlled ventilation and more precise titration of inspired oxygen.

These considerations are reinforced by reports of surgical fires associated with HFNO during head and neck procedures, particularly in patients undergoing superficial surgery under sedation [30]. The growing number of case reports describing ignition in oxygen-enriched fields emphasises the primary importance of patient safety and, consequently, the potential medicolegal implications of using HFNO in these settings. In common law negligence claims, liability may arise if an injured patient demonstrates that foreseeable harm was not mitigated by appropriate precautions, thereby constituting a breach of the expected standard of care. The use of supplemental oxygen, particularly at high concentrations, in proximity to an ignition source, may therefore be difficult to defend, in the opinion of our legal expert, where safer alternatives exist [36,37]. Consequently, clinicians should be able to clearly justify the decision to use HFNO in head and neck procedures, especially when lower FiO_2_ strategies or a secured airway could reduce the risk of fire.

Finally, clinicians should remain alert to the potential for preserved oxygen saturation to create false reassurance. HFNO enhances oxygenation but does not guarantee adequate ventilation, airway protection, or haemodynamic stability. Reports from adverse event databases, including webAIRS [38], describe cases of severe, unrecognised hypercarbia during HFNO-supported anaesthesia in which oxygenation was maintained but profound hypoventilation led to cardiac arrest or prolonged postoperative unconsciousness. These cases reinforce that adequate saturation does not equate to effective ventilation. Appropriate patient selection, vigilant monitoring of ventilation (not oxygenation alone), awareness of infection control, and readiness to escalate to definitive airway control remain central to safe practice.

In summary, HFNO is a valuable adjunct in contemporary anaesthesia, but its effectiveness depends on airway patency, and its use must be balanced against bleeding risk, infection hazards, anatomical considerations, aspiration potential, and the risk of oxygen-enriched fire, particularly in shared-airway and procedural sedation environments.

## 6. Environmental Impact and Sustainability Considerations

As the use of HFNO expands, it is also timely to consider its environmental impact, particularly in low-risk patients or in clinical scenarios where evidence of benefit is limited. Compared with COT devices, HFNO requires specialised consumables, active gas heating and humidification, higher oxygen flow rates, and greater electricity consumption. Together, these factors contribute to increased carbon emissions and greater volumes of single-use plastic waste.

Although published comprehensive life-cycle analyses remain limited, evidence suggests that HFNO has a substantially greater environmental footprint than COT. An Australian study [39], found that HFNO used for pre-oxygenation before tracheal intubation generated an additional 0.9 kg CO_2_e (carbon dioxide equivalents) per patient compared with facemask oxygen delivery, equivalent to driving approximately 3.3 km in a petrol-powered vehicle. This was likely a conservative estimate.

Further detailed carbon-footprinting data indicates that HFNO consumables have substantially higher embodied carbon than standard nasal cannulae alone, with HFNO nasal prongs associated with approximately 1.5-fold higher CO_2_e emissions and HFNO breathing circuits 8–9-fold higher emissions than standard nasal cannula systems. The preliminary, unpublished carbon-footprinting values were calculated by the Healthcare Carbon Lab using recognised life-cycle assessment methods, including Australian-specific life-cycle inventory data (AusLCI), with estimates dependent on modelling assumptions such as local electricity generation and waste management practices (personal communication, Scott McAlister, Healthcare Carbon Lab, The University of Melbourne).

The environmental impact of HFNO is further influenced by oxygen consumption. Medical oxygen production itself has a carbon footprint that varies with manufacturing processes and energy sources [40], with emissions increasing in proportion to oxygen flow rates and delivered FiO_2_. Healthcare is estimated to account for approximately 5% of global greenhouse gas emissions [41,42], with a substantial proportion of clinical care of limited or uncertain value [43].

In our view, HFNO should therefore be used selectively rather than as a default oxygenation strategy. Practical stewardship measures include reserving HFNO for patients and procedures with a clear clinical rationale; avoiding routine use in low-risk patients when COT is adequate; using the lowest effective flow and FiO_2_ once clinically appropriate; and discontinuing HFNO when it is no longer required. Selective use, careful titration, and avoidance of low-value therapy represent important opportunities to reduce environmental impact while maintaining high-quality perioperative care.

## 7. Clinical Applications

**Figure 3:**
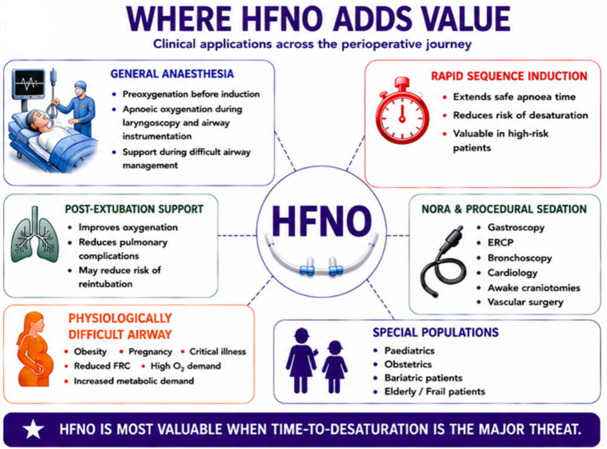
HFNO current uses with stronger evidence.

The clinical applications of HFNO span perioperative care, procedural sedation, NORA, shared-airway surgery and selected specialist settings (Figure 3). Because the evidence base varies substantially across these areas and continues to evolve, Table 2 provides a pragmatic summary of the relative strength of evidence, potential clinical role, limitations and practical recommendations before each area is considered in greater detail.

### 7.1. General Anaesthesia

Peroxygenation is the continuous delivery of oxygen throughout the entire period of airway management, including pre-induction, airway instrumentation, and the apnoeic phase, to maintain oxygenation and prolong safe apnoea time. This concept extends traditional approaches by integrating preoxygenation, which occurs before induction of anaesthesia to maximise oxygen reserves, and apnoeic oxygenation, which delivers oxygen during apnoea to maintain alveolar oxygen diffusion in the absence of ventilation. Peroxygenation, therefore, emphasises uninterrupted oxygen delivery across the entire airway management sequence and is particularly well facilitated by HFNO.

There is growing evidence supporting the utility of HFNO in achieving optimal preoxygenation [44]. A systematic review of 3914 patients and meta-analysis of various preoxygenation strategies found that HFNO in the head-up position was the most advantageous approach for prolonging safe apnoea time [3]. Administration of HFNO during the peri-induction period achieves peroxygenation, delivering oxygen both before the onset of apnoea and continuously throughout airway instrumentation [45,46,47]. The advantage of this approach has been underscored by the Difficult Airway Society (DAS) tracheal intubation guidelines, which advocate continuous oxygen delivery during laryngoscopy whilst ensuring a patent airway [48]. Similarly, the 2022 ASA Difficult Airway Guidelines emphasise the increased margin of safety afforded by continuous oxygen delivery before, during, and after intubation [49].

Evidence also supports the use of HFNO in anticipated difficult airway management due to either physiological compromise or anatomical factors [50]. These benefits have been demonstrated across diverse patient populations, including those with limited physiological reserve, thereby enabling the safer application of non-routine techniques often required in difficult airway management [45,51]. However, a recent systematic review and network meta-analysis reported that non-invasive positive pressure ventilation probably decreases the incidence of hypoxaemia during intubation more than HFNO in critically ill patients [52].

From a practical perspective, the Optiflow Switch (Fisher & Paykel Healthcare, Auckland, New Zealand) incorporates a flow-regulated pressure-relief valve and a compressible inflow to the nasal prongs, allowing interruption of nasal gas flow when a tight-fitting facemask is applied [53]. This facilitates a safe transition between positive pressure ventilation and HFNO. Practical recommendations for administering HFNO suggest using an FiO_2_ of 1.0 with the mouth closed for 3–5 min [54]. It has proposed an evidence-informed, opinion-based approach to determining flow rates for preoxygenation using HFNO [55]. In low-risk patients, HFNO ≥ 30 L min^−1^ may be used as an alternative to facemask ventilation with PEEP and/or pressure support. In high-risk patients (e.g., predicted difficult airway, rapid desaturation, or when facemask ventilation should ideally be minimised), HFNO ≥ 60 L min^−1^ is recommended as first-line therapy with the patient positioned 20–30° head-up, titrated to comfort, and continued throughout laryngoscopy. Of note, recent evidence has confirmed that arterial carbon dioxide accumulation during apnoea is minimally affected by HFNO administration [56].

#### 7.1.1. Rapid Sequence Induction

Lengthening of the safe apnoea window with HFNO has also been shown to reduce desaturation events during modified rapid-sequence intubation in non-elective patient [57]. Extension of safe apnoea time in this subset of patients is especially relevant when face-mask ventilation is relatively contraindicated due to concerns about gastric regurgitation [47]. A recent meta-analysis [58] included 6 studies and found that HFNO may offer advantages over face-mask ventilation during RSI in the emergency setting. However, this potential benefit must be weighed against concerns about gastric insufflation and increased intragastric pressure, which could increase the risk of regurgitation in unfasted patients, particularly those with a hiatus hernia [59]. Although case reports have raised this possibility [59], current evidence remains limited and inconclusive, and available physiological and clinical studies have not consistently demonstrated a clinically significant increase in regurgitation risk with HFNO. Consequently, while this risk should be considered, especially in high-risk patients, it appears likely to be very low based on current data [21,24,60,61].

#### 7.1.2. Awake Tracheal Intubation

Evidence is also accumulating that HFNO improves the safety of awake flexible bronchoscopic intubation and can be utilised to maintain oxygenation without interrupting airway access [54,62,63]. Some of the benefits may be attributed to HFNO widening the pharyngeal space, thereby improving the view of the glottis [64]. It may be especially useful if sedation is required to ensure patient cooperation. Likewise, its use has been reported during awake tracheostomy, but precautions may be required to ensure that a high FiO2 does not increase the risk of airway fire in this context [65,66].

#### 7.1.3. Tubeless Airway Surgery

Tubeless (“open”) airway surgery refers to laryngotracheal procedures performed without placement of a tracheal tube. Instead, the airway is “opened” using an ENT suspension laryngoscope. HFNO in the shared-airway setting offers several advantages compared with alternative oxygenation strategies during microlaryngoscopy (Table 3). Standard anaesthetic monitoring, including pulse oximetry, non-invasive blood pressure, electrocardiography, and capnography, should be applied. Because the airway remains tubeless, volatile anaesthetic agents cannot be reliably delivered. Anaesthesia is therefore typically maintained using total intravenous techniques, most commonly propofol administered via target-controlled infusion (TCI). Additional agents, such as a small midazolam bolus and a remifentanil infusion, may be used for anxiolysis and analgesia. Following induction of anaesthesia, airway patency is initially supported with jaw thrust until suspension laryngoscopy is established.

HFNO used during apnoea [68] under general anaesthesia and neuromuscular blockade has been termed Transnasal Humidified Rapid-Insufflation Ventilatory Exchange (THRIVE) [50]. This study included obese and stridulous patients and showed an extended safe apnoea time with a median (range) of 14 (5–65) min. However, subsequent work has shown that HFNO under apnoeic conditions does not effectively eliminate carbon dioxide. Carbon dioxide accumulation occurs at a relatively consistent rate across a wide range of flow rates, leading to progressive hypercapnia [69]. If prolonged, this may result in respiratory and metabolic acidosis, arrhythmias, and haemodynamic instability.

An alternative strategy involves using HFNO while maintaining spontaneous ventilation (HFNO-sv), also known as STRIVE H [69,70]. Careful titration of propofol TCI can allow spontaneous ventilation to be preserved even at deep levels of anaesthesia sufficient for jaw thrust, topicalisation of the vocal cords with lignocaine, suspension laryngoscopy, and surgery. Maintaining spontaneous respiration may provide an additional safety margin by limiting carbon dioxide accumulation and reducing the risk of significant respiratory acidosis compared with apnoeic HFNO techniques [70,71,72]. In patients with significant airway or respiratory compromise, HFNO-sv has been shown to maintain adequate oxygenation, ventilation, and airway patency [71]. The median (range) end-tidal carbon dioxide (ETCO_2_) was 6.8 (4.8–8.9) kPa, with a mean rate of CO_2_ rise substantially lower than that reported with apnoeic HFNO (0.03 vs. 0.15–0.6 kPa/min) [69,71].

HFNO-sv also facilitates dynamic airway assessment during microlaryngoscopy, allowing clinicians to distinguish between fixed and functional obstructive airway lesions and may be particularly valuable for lesions involving the supraglottic, subglottic, and tracheal region [70,71]. Despite these advantages, complications can occur, including apnoea, hypoxaemia, or intolerance of suspension laryngoscopy [70]. During airway balloon dilatation, temporary suppression of respiration may be required; this can be achieved with a small bolus of remifentanil to minimise the risk of negative-pressure pulmonary oedema.

During suspension laryngoscopy, the mouth remains open, reducing the continuous positive airway pressure effect of HFNO. With the mouth closed, increasing HFNO flow can generate approximately 0.7–1 cmH_2_O of airway pressure for every 10 L min^−1^ increase in flow [6]. With an open mouth, however, this effect is markedly reduced (0.01 cmH_2_O per 10 min) [45,73].

Monitoring carbon dioxide during tubeless airway surgery remains challenging. During apnoeic HFNO, conventional capnography is unavailable. Even during spontaneous ventilation, reliable capnography can be difficult to obtain, and adjunctive monitoring such as ECG-derived respiration may be useful. Alternative methods for tracking carbon dioxide accumulation include periodic arterial blood gas analysis, intermittent sampling via a subglottic catheter, or transcutaneous CO_2_ monitoring.

The use of HFNO during airway surgery involving diathermy or laser remains controversial due to concerns about airway fire. Current practices vary and include temporary cessation of HFNO, reduction in inspired oxygen concentration, maintenance of high FiO_2_, or reduction in flow rates [30,74].

Although multiple observational series have reported the use of HFNO at an FiO_2_ of 1.0 during tubeless laser airway surgery [30], these findings are based on level 4 evidence and do not provide robust assurance of safety [75]. Other experts have therefore highlighted the absence of high-quality data to support this practice. A more conservative, safer approach is to avoid HFNO during laser or diathermy use, except in exceptional circumstances [30]. Such an approach aligns more closely with professional guidance and manufacturers’ instructions for use. Consequently, from a medicolegal perspective, the use of HFNO at high inspired oxygen concentrations during laser or diathermy airway surgery may be difficult to justify or defend.

### 7.2. Extubation and Postoperative Use of HFNO

Postoperative hypoxaemia and pulmonary complications, including atelectasis and pneumonia, are more common in high-risk patients and following prolonged general anaesthesia. HFNO has emerged as an alternative to COT after extubation. Its physiological benefits include providing low-level PEEP and reducing the work of breathing.

HFNO is typically initiated immediately after extubation in the OR and continued into the recovery period. In some studies, however, extubation and initiation of HFNO occurred in higher-acuity settings, such as high-dependency units or intensive care units (ICUs).

A 2025 meta-analysis [2] involving over 1800 patients demonstrated improved oxygenation and reduced pulmonary complications, particularly in non-cardiothoracic surgical populations. Although this analysis included a heterogeneous group of 17 relatively small randomised controlled trials, the findings suggest that larger, well-designed studies with standardised protocols would likely strengthen the evidence base.

Earlier evidence also supports these findings. A 2016 study of 184 critically ill surgical patients extubated in the ICU showed that immediate application of HFNO reduced reintubation rates [76]. This was reinforced by a 2019 meta-analysis of 10 studies (1327 patients), which reported a significant reduction in the risk of reintubation (relative risk, 0.38) with HFNO compared with COT [77]. Similarly, HFNO has shown benefit in high-risk surgical populations, such as patients undergoing oesophageal cancer surgery, with reduced postoperative pneumonia compared with COT [78].

Although large-scale trials remain limited, the available evidence suggests that using HFNO immediately after extubation in non-cardiothoracic surgical patients may reduce the risk of reintubation and improve postoperative respiratory outcomes.

### 7.3. Non-Operating-Room Anaesthesia

Historical analyses from the ASA Closed Claims Project demonstrate that adverse events in NORA [79,80] are predominantly respiratory, with hypoxaemia and airway obstruction accounting for the majority of severe outcomes. These events occur most commonly during monitored anaesthesia care (MAC) rather than general anaesthesia. Mortality has been higher in NORA compared with operating room practice, and aspiration events are more frequent [79]. Many complications are associated with the absence of capnography. Although gastrointestinal endoscopy accounts for the largest number of cases, cardiology and radiology are overrepresented relative to their procedural volumes, suggesting increased relative risk [79]. Patients undergoing NORA frequently present with complex clinical profiles, including advanced age, higher ASA status, and comorbidities such as obstructive sleep apnoea and cardiorespiratory disease, increasing susceptibility to sedation-related respiratory compromise. These risks are further compounded by the procedural environment, as NORA is delivered in remote locations outside the operating theatre, where logistical challenges, limited airway access, and variable resources may impact the timely recognition and management of adverse events [81].

Updated analyses confirm that while respiratory events remain central, adverse outcomes increasingly reflect interactions between physiological and human factors [80]. Situational awareness errors contribute to nearly three-quarters of major adverse events, and communication failures occur in over 40% [80]. HFNO may mitigate hypoxaemia by improving oxygenation [82]; however, it is important to note that it does not address hypoventilation, airway obstruction, aspiration risk, or human factors. It should therefore be viewed as part of a broader, system-wide airway safety strategy.

#### 7.3.1. Gastroenterology

Upper gastrointestinal endoscopy and colonoscopy are common NORA procedures that are frequently complicated by hypoxaemia [83]. This is driven by sedation-induced respiratory depression and loss of airway tone, exacerbated in gastroscopy by the shared airway. Capnography improves the detection of hypoventilation compared with pulse oximetry alone [84].

HFNO reduces hypoxaemia across a spectrum of risk. In low-risk patients, HFNO has been shown to almost eliminate hypoxaemia during gastroscopy [85]. In higher-risk populations, including those with comorbid disease, the ODEPHI trial demonstrated a reduction in hypoxaemia from 33.5% to 9.4% with fewer airway interventions [86]. In patients with obesity, large multicentre data confirm substantial reductions in hypoxaemia and severe hypoxia [87]. Smaller randomised trials and reviews [85,86,88,89,90] support these findings, demonstrating reduced desaturation and fewer airway interventions, although an increased incidence of apnoea has been observed [89].

Endoscopic retrograde cholangiopancreatography (ERCP) represents a higher-risk NORA procedure due to the combination of deep sedation, prone positioning, and restricted airway access. These factors increase the risk of hypoxaemia and complicate airway rescue, particularly in elderly and comorbid patients. In a randomised trial, HFNO increased minimum oxygen saturation and reduced airway-related procedural interruptions compared with COT [91]. Similar findings have been demonstrated in elderly patients, in whom HFNO significantly reduced hypoxaemia and improved oxygen saturation during propofol sedation [92].

#### 7.3.2. Respiratory

Flexible bronchoscopy and endobronchial ultrasound (EBUS) are associated with a high incidence of hypoxaemia during procedural sedation, particularly in patients with underlying respiratory disease or reduced physiological reserve. Airway access is shared and often limited, and desaturation may lead to procedural interruption and require airway intervention.

HFNO has been shown to improve oxygenation and reduce hypoxaemic events during bronchoscopy. Two meta-analyses of randomised controlled trials demonstrate consistent reductions in desaturation, improvements in oxygenation, and fewer procedural interruptions compared with low-flow oxygen therapy [93,94]. These findings are supported by individual trials [95,96]. In a randomised controlled study of lung transplant recipients undergoing bronchoscopy, HFNO significantly reduced both mild and severe desaturation and eliminated procedural interruptions [97]. Collectively, these data support the role of HFNO in improving procedural stability and reducing the need for airway interventions.

However, HFNO does not provide ventilatory support and may mask hypoventilation by maintaining oxygen saturation despite rising carbon dioxide levels. When compared with non-invasive ventilation (NIV), the relative benefit remains uncertain, with NIV potentially offering superior support in patients with severe baseline hypoxaemia. HFNO should therefore be considered an adjunct to improve oxygenation, used alongside appropriate monitoring and readiness to escalate airway or ventilatory support.

#### 7.3.3. Cardiology and Structural Heart Procedures

Non-operating-room anaesthesia in cardiology includes procedures such as cardiac implantable electronic device (CIED) insertion, electrophysiology studies, and structural interventions, including transcatheter aortic valve implantation (TAVI). These are increasingly performed under procedural or conscious sedation in environments where airway access may be restricted by imaging equipment and patient positioning. Patients are often elderly with significant cardiovascular and respiratory comorbidity, increasing the risk of hypoxaemia and sedation-related complications.

Evidence for HFNO in this setting is heterogeneous but evolving. In adults undergoing CIED procedures, a randomised controlled trial found no significant difference in ventilation or oxygen desaturation compared with facemask oxygen but reported a higher incidence of minor sedation-related adverse events in the HFNO group [98]. Earlier data in TAVI similarly showed no improvement in arterial oxygenation, although reductions in desaturation and improved patient comfort were observed [99]. More recent evidence suggests a clearer benefit. The HIGH-OXY-TAVR randomised trial demonstrated that HFNO significantly reduced desaturation events (20% vs. 51%) and improved arterial oxygenation compared with COT [100]. In contrast, benefits have also been demonstrated in selected high-risk populations, including paediatric patients with congenital heart disease, in whom HFNO improved oxygenation and reduced the need for assisted ventilation [101].

Importantly, many cardiology procedures, particularly CIED insertion, involve the use of diathermy and introduces the potential risk of procedural fire in oxygen-enriched environments.

Overall, the effectiveness of HFNO in cardiology procedures appears to be context- and population-dependent, with emerging evidence supporting its use in selected high-risk settings.

#### 7.3.4. Vascular and Endovascular Procedures

Evidence for HFNO in vascular and endovascular procedures remains limited. These procedures are typically performed under deep sedation in patients with significant comorbidity, where hypoxaemia and airway obstruction are common, and airway access may be restricted. In a small, randomised trial of patients undergoing endovascular surgery under propofol sedation, HFNO reduced desaturation, airway obstruction, and the need for airway manoeuvres compared with COT, without improving arterial blood gas parameters [102]. While these findings suggest a potential role for HFNO in improving procedural stability, they are derived from a single, small study.

#### 7.3.5. Neurosurgery

Within neurosurgery, awake craniotomy represents a specific situation in which HFNO may be particularly advantageous. Unlike conventional low-flow nasal prongs, HFNO delivers warmed, humidified gas at high flow rates, providing more reliable oxygen delivery, dead-space washout, and a degree of low-level positive airway pressure. This may help reduce airway obstruction while preserving speech, language, and memory testing during often prolonged procedures. HFNO also differs from asleep–awake–asleep techniques that rely on supraglottic airways or tracheal tubes, as it may avoid airway instrumentation, coughing, bucking, and airway trauma. Its humidification and soft nasal interface may improve airway comfort over long-duration surgery. Case reports describe successful use in obese and OSA patients [103,104,105], while prospective data suggest that HFNO reduces airway obstruction and injury compared with nasopharyngeal airway use, without worsening brain relaxation or increasing gastric antral volume on ultrasound [106]. Careful sedation titration and monitoring for hypoventilation or hypercapnia remains essential.

#### 7.3.6. Interventional Radiology

Interventional radiology is a related high-risk NORA environment with a disproportionately high incidence of adverse events reported in large datasets [79,80]. Procedures are frequently performed under deep sedation in remote locations with restricted airway access and variable monitoring conditions. Despite these risks and the increasing clinical adoption of HFNO, there is a notable absence of published evidence evaluating its use in this setting. This represents an important gap in the literature and an area for future research.

#### 7.3.7. Dental

Evidence for HFNO in this setting remains limited. Small randomised trials demonstrate improved oxygenation, reduced hypoxaemia, and fewer airway interventions compared with COT [107,108]. These findings are consistent across adult and paediatric populations, including high-risk groups such as obese patients [108,109]. A scoping review of the available literature similarly supports these findings but highlights the small number and size of included studies [110].

Importantly, dental procedures frequently involve electrocautery or other ignition sources and may increase the risk of fire. HFNO may offer practical advantages due to its unobstructive nasal interface; however, given the limited and heterogeneous evidence base, its use should be individualised and accompanied by vigilant monitoring and readiness to manage the airway.

#### 7.3.8. Plastic and Facial Surgery

Published evidence on HFNO in plastic and facial surgery is extremely limited and largely confined to fire-related risks [27,28,29,30,31,32,33,34,35]. Supplemental oxygen can accumulate beneath surgical drapes (“oxygen pooling”), creating an oxygen-enriched environment that markedly increases combustibility in the presence of ignition sources such as electrocautery [111]. Given this, high FiO_2_ oxygen delivery—including HFNO—should be avoided during facial surgery when ignition sources are used.

#### 7.3.9. Assisted Reproductive Procedures

Assisted reproductive treatment cycles continue to increase across Australia and New Zealand [112], resulting in a parallel rise in anaesthesia-supported procedures, most commonly transvaginal ultrasound-guided oocyte retrieval performed under deep sedation. These procedures are typically undertaken in the lithotomy position, often with Trendelenburg tilt, and are associated with frequent sedation-related respiratory events including hypoventilation, airway obstruction and oxygen desaturation.

HFNO is increasingly being adopted in this setting. Evidence from the broader procedural sedation literature demonstrates reductions in hypoxaemia, airway interventions and procedural interruptions compared with conventional oxygen delivery strategies. Although evidence specific to oocyte retrieval remains limited, these findings are consistent with growing clinical experience within high-volume fertility services.

The physiological advantages of HFNO may be particularly relevant in this setting. Deep sedation during oocyte retrieval commonly results in hypoventilation, upper airway obstruction and intermittent apnoea, risks that may be exacerbated by Trendelenburg positioning, advanced maternal age, cardiorespiratory comorbidity and obesity [113,114]. By providing high inspired oxygen concentrations, nasopharyngeal dead-space washout, and low-level positive airway pressure, HFNO may improve oxygen reserve and reduce the frequency of sedation-related respiratory compromise.

Beyond physiological benefits, HFNO may offer practical advantages in assisted reproductive services where procedural efficiency and uninterrupted operating conditions are important. Improved oxygenation may facilitate stable deep sedation with fewer airway interventions or procedural pauses, potentially improving workflow in high-throughput environments. Some high-volume fertility centres have incorporated HFNO into practice for selected high-risk patients, including those with anterior mediastinal masses undergoing fertility preservation prior to oncological treatment, where its dynamic CPAP effects may help reduce airway collapse.

### 7.4. The Physiologically Difficult Airway

The physiologically difficult airway (PDA) has recently been recognised as an important non-anatomical risk factor in surgical patients undergoing tracheal intubation, associated with an increased risk of hypoxaemia and cardiac arrest [115,116]. It is typically encountered in patients with significant physiological derangement, including those with obesity, pregnancy, critical illness, or right heart failure. These groups are widely considered to be at the highest risk during intubation and therefore require enhanced planning, resources, and experienced personnel [116].

Intubation in patients with a PDA is associated with a higher likelihood of rapid and profound desaturation during apnoea following induction of anaesthesia. This is largely attributable to reduced oxygen reserve, decreased functional residual capacity, and/or haemodynamic instability [115,116].

An international guideline on the management of patients with a PDA in the OR has recently been published by an expert group from the Society of Critical Care Anaesthesiologists (SOCCA) [116]. This guideline was developed using a Delphi consensus process involving 40 international airway experts and produced 53 recommendations to support safer tracheal intubation practices in this high-risk population.

One key recommendation states: “Apnoeic oxygenation (i.e., oxygen delivery during apnoea) using HFNO is an acceptable technique to minimise oxygen desaturation during tracheal intubation in patients with a physiologically difficult airway.”

This recommendation is largely based on studies conducted in critically unwell patient populations. The PROTRACH study (2019) demonstrated that HFNO for apnoeic oxygenation during intubation reduced the incidence of oxygen desaturation below 95% compared with bag-mask ventilation (12% vs. 23%) [117]. The FLORALI-2 trial evaluated 313 patients with severe respiratory failure across 28 hospitals in France and compared non-invasive ventilation (NIV) with HFNO for preoxygenation [118]. No significant difference in the rates of severe hypoxaemia was observed, although a subgroup analysis suggested a potential benefit of NIV.

Overall, the evidence supporting HFNO as a preoxygenation strategy in patients with a PDA is less robust than that for NIV, as highlighted by the PERIOX study published in the New England Journal of Medicine in 2024 [119]. However, the choice between HFNO and NIV also depends on the clinical setting and whether the dominant problem is oxygenation, ventilation, or alveolar recruitment. In many urgent anaesthetic settings, HFNO may be the more practical preoxygenation strategy, as NIV may not already be established and may be difficult to initiate without delaying airway management. An important practical advantage of HFNO is that it can be continued throughout laryngoscopy, which may be particularly beneficial when airway instrumentation is anticipated to be difficult.

In contrast, in ICU patients already established on NIV, particularly those with hypoventilation, hypercapnia, atelectasis, shunt physiology, obesity hypoventilation, or cardiogenic pulmonary oedema, NIV is often preferable for preoxygenation. In such cases, and where suitable equipment is available, adding an HFNO system with compressible nasal cannula segments and integrated flow-diverter mechanisms to NIV as an escalation step may be prudent. This allows NIV to be used for preoxygenation while preserving the option to transition rapidly to HFNO for airway instrumentation and apnoeic oxygenation. This approach reflects expert opinion and pragmatic clinical practice rather than definitive comparative evidence.

### 7.5. HFNO in Patients with Reduced Physiological Reserve

#### 7.5.1. Patients Living with Obesity

Obesity is increasingly recognised as a chronic, systemic disease characterised by excess adiposity and associated physiological dysfunction [120]. Although obesity is traditionally defined as body mass index (BMI) ≥ 30 kg m^−2^, [121] contemporary approaches advocate combining BMI with additional anthropometric measures, such as waist-to-hip ratio, to better reflect obesity-related health risk [120].

The respiratory consequences of obesity are multifactorial and clinically significant. Excess adiposity impairs respiratory mechanics through reductions in static and dynamic lung volumes, airway narrowing, chronic micro-atelectasis, and increased work of breathing [122,123,124]. Functional residual capacity is reduced, oxygen consumption increased, and airway closure may occur during normal tidal ventilation, all of which predispose patients to rapid oxygen desaturation during apnoea. These physiological changes contribute to increased perioperative respiratory risk.

HFNO may mitigate several of these respiratory derangements in patients living with obesity. Proposed mechanisms include the creation of an upper airway oxygen reservoir, generation of low levels of positive airway pressure, improved lung compliance and reduced work of breathing [5,125,126]. Together, these effects may enhance alveolar recruitment and oxygen reserve, thereby improving pre-oxygenation and maintenance of oxygenation during periods of apnoea or respiratory compromise.

Contemporary airway management guidelines for patients living with obesity [127] recommend HFNO as a first-line strategy for both pre-oxygenation and apnoeic oxygenation. This recommendation reflects the heightened risk of rapid desaturation in this population, driven by impaired respiratory mechanics, reduced functional residual capacity and increased oxygen demand. Clinical studies suggest that HFNO may reduce oxygen desaturation during apnea [128,129] compared with conventional oxygen delivery methods, such as the Hudson facemask [130]. Prolonged apnoeic oxygenation times have also been reported [128], with one study demonstrating maintenance of acceptable oxygen saturation for up to 18 min in the majority of patients with morbid obesity [130]. These findings support the concept that HFNO may extend safe apnoea time and provide a greater margin of safety during airway management and tracheal intubation.

Evidence regarding postoperative HFNO in patients living with obesity is heterogeneous but generally encouraging [131,132]. The timing of HFNO initiation may be important. In bariatric surgery, early application of HFNO prior to extubation and its continuation into the immediate postoperative period have been associated with reduced postoperative hypoxaemia [131]. In contrast, another study found no significant difference compared with COT, possibly due to alveolar derecruitment before HFNO initiation [132].

HFNO has also been recommended following cardiac and thoracic surgery in patients living with obesity [133]. Studies in patients undergoing cardiopulmonary bypass have demonstrated improved oxygenation, lower atelectasis scores, reduced reintubation rates and improved patient comfort with HFNO [134,135]. However, evidence remains inconsistent, with some meta-analyses demonstrating no significant differences in atelectasis, dyspnoea, reintubation, or intensive care unit length of stay, although interpretation is limited by the small number of heterogeneous studies [136].

#### 7.5.2. Obstetrics

The rationale for HFNO in obstetric general anaesthesia is compelling [137]. Pregnancy-related physiological changes, mediated by progesterone, oestrogen and relaxin, affect airway characteristics, reduce functional residual capacity, increase metabolic demand and heighten the risk of aspiration, all of which predispose parturients to rapid desaturation during apnoea [138]. Difficult and failed intubation are more common in obstetric practice, and the consequences for both mother and fetus may be critical [139,140,141,142]. Although neuraxial anaesthesia predominates, general anaesthesia remains necessary in selected circumstances in which optimisation of oxygen reserves is essential. These risks are further amplified by common comorbidities such as obesity, and reflect a combination of anatomical, physiological and human factors.

The theoretical benefit of HFNO in extending safe apnoea time has been supported by computational simulation studies using validated pregnancy models, including scenarios that incorporate labour, obesity, and other comorbidities [143]. Given the established benefit of HFNO in non-pregnant adults, it is plausible that similar physiological advantages translate to pregnant patients in vivo. However, demonstrating an effect on clinical outcomes such as time to desaturation, incidence of hypoxaemia, failed tracheal intubation, or maternal complications remains challenging. Studies designed to delay intubation or oxygenation in order to measure these end points would expose already high-risk patients and their unborn babies to unnecessary and unjustifiable risk.

Available heterogeneous quantitative evidence suggests that HFNO may offer advantages for pre-oxygenation in pregnant patients [144,145,146]. End-tidal oxygen concentration remains the guideline-recommended surrogate for pre-oxygenation efficacy. In a randomised controlled trial of term pregnant women, 71% of participants achieved an adequate end-tidal oxygen concentration of ≥90% with HFNO, compared with 44% using conventional facemask oxygen [145]. Other trials have demonstrated higher arterial oxygen partial pressures with HFNO than with a face mask, particularly in patients living with obesity [146,147]. This suggests that HFNO may improve oxygen reserves beyond what conventional end-tidal oxygen measurements alone captures.

Practical considerations are also important. HFNO systems require preparation separate from standard airway equipment, although increasing familiarity and routine availability may reduce this barrier. HFNO pre-oxygenation is feasible in anxious or unwell patients, particularly in emergency settings. Devices such as Optiflow Switch™ may further improve usability by allowing expired end-tidal oxygen measurement before induction [15] and enabling transition between HFNO and facemask oxygenation without removing the nasal cannulae. This facilitates early oxygen delivery on arrival in theatre, continuation during airway instrumentation, and gentle bag-mask ventilation while awaiting neuromuscular blockade, in line with guideline recommendations. A hands-free approach may also improve patient experience by preserving communication with the anaesthetist, midwife and wider team, rather than relying solely on a tight-fitting facemask in an already stressful situation. At present, HFNO is best regarded as a useful adjunct to established facemask-based pre-oxygenation strategies in obstetric general anaesthesia.

#### 7.5.3. Paediatrics

In paediatric anaesthesia, HFNO should not be viewed simply as an oxygen-delivery tool but as a dynamic, physiologically driven airway safety measure. Its clinical value lies in its ability to modify respiratory physiology to mitigate the rapid desaturation that characterises anaesthesia in children. This is particularly relevant at points where children, especially infants and neonates, are least tolerant of ventilation interruptions and therefore most vulnerable to sudden oxygen desaturation, namely during airway instrumentation and shared airway surgery. In this setting, HFNO offers more than oxygen supplementation alone [148].

HFNO delivers heated, humidified gas at flow rates that exceed spontaneous inspiratory demand, commonly around 1–2 L kg^−1^ min^−1^, thereby effectively converting the upper airway into a functional oxygen reservoir and increasing the margin of safety during periods of reduced or absent ventilation [11,149]. One of its principal mechanisms is nasopharyngeal dead space washout, whereby exhaled carbon dioxide is cleared and replaced with oxygen-rich gas. This helps delay hypoxaemia by optimising the oxygen available for apnoeic oxygenation via a ventilatory mass flow, in which the atmospheric pressure gradient facilitates oxygen movement into the alveoli, even during severe apnoea [11]. HFNO also generates a small amount of positive airway pressure, which may help open the compliant paediatric airway and maintain FRC. This pneumatic effect may decrease atelectatic change and lessen the physiological instability associated with airway manipulation and interrupted ventilation. Delivering gas that has been conditioned to body temperature and fully humidified helps maintain mucosal integrity and reduces airway irritation and reflex bronchospasm [150].

Evidence from more challenging shared airway settings is also encouraging. In children undergoing surgery for juvenile-onset recurrent respiratory papillomatosis, HFNO improved oxygenation, prolonged apnoea time, and slowed carbon dioxide accumulation, suggesting value in selected high-risk airway cases where maintaining unobstructed airway access is essential [151]. More recently, a meta-analysis found that perioperative HFNO in children was associated with reduced desaturation, higher minimum oxygen saturation, and fewer rescue airway interventions compared with conventional oxygen strategies, although heterogeneity across studies remains substantial [152].

In paediatric anaesthesia, the use of HFNO therefore represents more than the provision of high-flow oxygen; it is a proactive strategy for maintaining physiological stability. Its value lies in supporting management of the at-risk airway by prioritising preservation of oxygenation from the outset rather than reacting to deterioration. This approach creates a longer, safer apnoeic window, which is particularly important for children with limited oxygen reserve and higher metabolic demands. It also provides additional time, preserves surgical access, and reduces the risk of physiological collapse during critical airway moments. Looking ahead, the use of HFNO in paediatric anaesthesia is likely to become increasingly targeted and protocol-driven, tailored to specific procedures and individual patient risk profiles.

## 8. Future Research

Further research is required to define where HFNO provides meaningful clinical benefit, where its use is neutral, and where it may add cost, complexity, risk, or environmental burden without clear advantage. Future studies should prioritise clinically relevant outcomes beyond oxygen saturation alone, including hypoxaemia, hypercapnia, airway interventions, escalation to general anaesthesia, reintubation, pulmonary complications, unplanned admission, patient safety events, and procedural success. Priority areas include HFNO use during procedural sedation, NORA, shared-airway surgery, induction and extubation, and in high-risk patient groups such as those with obesity, obstructive sleep apnoea, pregnancy, cardiorespiratory disease, or physiologically difficult airways. Standardised reporting of flow rates, FiO_2_ targets, sedation depth, patient positioning, monitoring methods, rescue interventions, and duration of HFNO use would improve comparison between studies and strengthen future guidance.

Safety and monitoring require particular attention. HFNO may preserve oxygenation despite inadequate ventilation, creating a risk of delayed recognition of hypoventilation, airway obstruction, or apnoea. Further work is needed to evaluate the effectiveness of ventilation monitoring during HFNO use, including capnography, transcutaneous carbon dioxide monitoring, and emerging respiratory monitoring technologies. Environmental and health economic outcomes should also be incorporated into future studies. Assessment of oxygen consumption, consumables, electricity use, waste generation, carbon footprint, cost-effectiveness, and implementation requirements will be essential for guiding the appropriate, evidence-based, and sustainable use of HFNO in contemporary anaesthetic practice.

## 9. Evolving Applications and Future Perspectives

Potential directions in HFNO are likely to focus on more individualised, physiology-guided applications rather than routine escalation of oxygen delivery. Important evidence gaps remain regarding optimal patient selection, flow rates, FiO_2_ targets, and integration with non-invasive ventilation. Future research should prioritise clinically meaningful outcomes beyond oxygen saturation alone, including hypoxaemia, reintubation, pulmonary complications, and patient safety. Additional work is needed in high-risk settings such as NORA, shared-airway surgery, and procedural sedation, where human factors and systems issues remain important contributors to adverse events. Ultimately, the future role of HFNO will depend not simply on its ability to improve oxygenation, but on how effectively it can be integrated into broader physiology-centred airway safety strategies.

## 10. Conclusions

HFNO has become an important adjunct in contemporary anaesthesia and airway management, with expanding applications across the operating room, remote and non-operating-room environments, and critical care settings. Its physiological benefits, including improved oxygen delivery, apnoeic oxygenation, dead-space washout, and low-level positive airway pressure, can enhance oxygen reserve and prolong safe apnoea time in selected patients. These advantages are particularly relevant in patients with reduced physiological reserve and in shared-airway or sedation-based procedures, where the risk of hypoxaemia is increased.

However, HFNO should not be viewed simply as a high-concentration oxygen delivery device or a substitute for definitive airway management. It does not prevent hypercapnia, aspiration, airway obstruction, or human-factor related complications, and may create false reassurance when oxygen saturation is preserved despite inadequate ventilation. Its use also raises important safety considerations, particularly regarding the risk of airway fire in oxygen-enriched environments.

**Figure 4:**
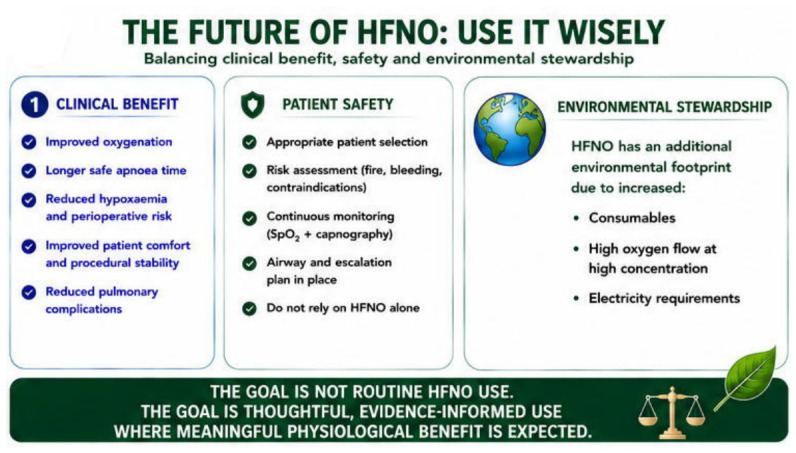
HFNO use considerations.

Ultimately, HFNO represents a significant advance in contemporary airway management, providing clinicians with an additional tool to improve oxygenation and extend the margin of safety during anaesthesia and procedural sedation. However, its benefits must be balanced against recognised limitations, safety considerations, and environmental costs (Figure 4). The goal should not be routine use, but thoughtful, evidence-informed application in patients and clinical scenarios where meaningful physiological or patient-centred benefit is expected.

## Figures and Tables

**Table 1:** Comparison of conventional oxygen therapy (COT), high-flow nasal oxygen (HFNO) and non-invasive ventilation (NIV).

Feature	Conventional Oxygen Therapy (COT)	High-Flow Nasal Oxygen (HFNO)	Non-Invasive Ventilation (NIV)
Primary role	Supplemental oxygen	High-flow oxygenation and apnoeic oxygenation	Oxygenation plus ventilatory support
Flow/pressure	Low to moderate flow; minimal pressure	High flow; low-level positive airway pressure	Positive pressure ventilatory support
FiO_2_ reliability	Variable, affected by inspiratory flow and entrainment	More reliable at high flows	Usually reliable with adequate mask seal
Dead-space washout	Minimal	Present	Variable
Humidification	Usually absent or limited	Heated humidification	Variable, depending on circuit/device
Ventilatory support	No	Limited	Yes
Airway protection	No	No	No
Procedural access/tolerance	Usually well tolerated	Usually well tolerated; preserves oral access	Mask interface may limit access and tolerance
Main limitation	May be inadequate in high-risk patients	Does not secure the airway or reliably treat hypoventilation	Requires seal, cooperation, monitoring and an appropriate interface

**Table 2:** Clinical applications of HFNO: evidence strength, potential role, limitations and practical recommendations.

Clinical Setting	Evidence Strength	Potential Role Advantages	Limitations/Cautions	Practical Recommendation
Preoxygenation before induction	Moderate to strong in selected patients	Improves oxygen delivery; may prolong safe apnoea time	Less ventilatory support than NIV; not ideal where severe hypercapnia or ventilatory failure is present	Consider in patients at risk of desaturation, particularly where apnoeic oxygenation may be useful
Physiologically difficult airway	Evolving	Can continue during laryngoscopy; supports apnoeic oxygenation and maintains airway access	Does not provide active ventilation; NIV may be preferable in ICU patients already established on NIV or where ventilation/recruitment is required	Use according to the dominant problem: oxygenation versus ventilation/recruitment
Apnoeic oxygenation/THRIVE	Moderate	May delay desaturation during airway instrumentation or shared-airway procedures	CO_2_ rises during true apnoea; not a substitute for ventilation	Useful adjunct when difficult, prolonged, or shared-airway instrumentation is anticipated
Tubeless airway surgery	Moderate	Maintains oxygenation while preserving surgical access	Hypercapnia, fire risk, limited airway access, and need for rescue planning	Use with careful case selection, monitoring, communication, and escalation planning
Extubation	Moderate	May reduce post-extubation hypoxaemia; often better tolerated than NIV	NIV may be needed for hypercapnia or ventilatory failure	Consider after extubation in selected high-risk patients; escalate if ventilation is inadequate
Procedural sedation NORA	Evolving	May reduce hypoxaemic episodes during sedation	Does not treat hypoventilation, airway obstruction, aspiration risk, or human-factor issues	Consider in higher-risk patients or longer procedures where oxygenation risk is increased; maintain capnography and rescue planning
GI endoscopy/ERCP	Moderate	May reduce hypoxaemic episodes during deep sedation or prolonged procedures	Does not treat hypoventilation, airway obstruction, aspiration risk, or human-factor issues	Consider in high-risk or prolonged procedures
Bronchoscopy	Moderate/evolving	Supports oxygenation during airway instrumentation	Shared airway; CO_2_ retention possible; airway access may be limited and potential difficult physiological airway	Useful in patients with close monitoring
Cardiology procedures	Evolving; mixed evidence	May reduce hypoxaemic episodes during prolonged sedation or supine procedures; may improve procedural tolerance	Does not treat hypoventilation, airway obstruction, aspiration risk, or human-factor issues important; evidence remains procedure-specific	Consider selectively in higher-risk patients or prolonged procedures where hypoxaemia is likely; avoid routine use where conventional oxygen is adequate
Vascular	Evolving	May support oxygenation during prolonged supine procedures, regional anaesthesia with sedation, or high-risk patients with limited physiological reserve	Does not treat hypoventilation, aspiration risk, airway obstruction, or procedural access challenges	Consider selectively in higher-risk patients, prolonged procedures, or sedation/regional anaesthesia cases where hypoxaemia risk is increased
Interventional radiology	No direct evidence	May support oxygenation in selected prolonged or high-risk procedures	Evidence is extrapolated from other NORA/procedural sedation settings; airway access and rescue may be challenging	Consider where there is a clear clinical rationale and increased desaturation risk.
Assisted reproductive procedures	No direct evidence	May support oxygenation during sedation in selected higher-risk patients	Evidence is extrapolated from other procedural sedation/NORA settings; many procedures are short and low risk; benefit is likely patient- and context-specific	Consider where there is a clear clinical rationale and increased desaturation risk
Awake craniotomy	Limited	May improve oxygenation and comfort during prolonged awake procedures	Patient tolerance, airway access, procedure duration, neurological testing, and sedation depth remain important	Consider in patients having prolonged awake craniotomy procedures
Obesity/OSA	Moderate/evolving	May improve oxygenation and prolong desaturation time	NIV may be preferable if hypoventilation, hypercapnia, or recruitment is required	Consider for oxygenation and apnoeic oxygenation; use NIV when ventilatory support is required
Pregnancy	Limited	Potential benefit where rapid desaturation risk is high	Aspiration risk, urgency, and definitive airway strategy remain central	Adjunct only; does not replace RSI planning or definitive airway management
Paediatrics	Limited	May improve oxygenation in selected settings	Age, size, tolerance, flow selection, and gastric insufflation concerns	Use selectively with paediatric expertise and appropriate monitoring
Procedures with high fire risk	Case reports, safety guidance and expert consensus	May provide oxygenation while preserving access	Oxygen enrichment near ignition sources; catastrophic but preventable fire risk	Avoid or minimise HFNO near ignition sources; use the lowest effective FiO_2_ or consider a secured airway when appropriate

**Table 3:** Advantages and disadvantages of HFNO in microlaryngoscopy surgery.

Oxygenation Methods	Advantages	Disadvantages
Microlaryngoscopy tube	Familiarity. Ventilation more controlled (less desaturation, hypercarbia and airway interventions). No entrainment of air. Capnography available. Prevent aspiration.	Difficult/impossible to place in difficult airways. Restricted surgical access. Fuel for airway fire.
Oxygen insufflation via suspension laryngoscope port	Simple and inexpensive.	Entrainment of air. Not warm or humidified gases causing tracheal mucociliary damage.
HFNO	Easy to use. Familiarity. More commonly available. Used in awake or anaesthetised patients, and also in apnoeic or spontaneously ventilating patients. Tubeless—optimal surgical access. HFNO-sv beneficial in ball-valve obstructing lesions or airway foreign bodies.	Entrainment of air. Various HFNO contraindications: Nasal obstruction. Recent nasal surgery. Epistaxis. Contraindicated during diathermy or laser. Base of skull fracture. Apnoeic HFNO in conditions where hypercarbia may have adverse consequences.
Low-flow transglottic tracheal catheter [67]	Simple and inexpensive.	Lack of familiarity. Inadequate surgical access. Not warm or humidified gases causing tracheal mucociliary damage.
Jet ventilation Supraglottic via suspension laryngoscope Translaryngeal via cannula Subglottic catheter	Safe in experienced hands. Less CO_2_ accumulation than HFNO. Can deliver warm and humidified gas. High-frequency ventilators Automated. Provided CPAP/PEEP. Avoid gas trapping.	Entrainment of air. High-frequency machine—expensive and complex. Lack of familiarity. Gas trapping in obstructing lesions. Barotrauma. Translaryngeal catheter—invasive, kinking Subglottic catheter—restricted surgical access.

## Data Availability

No new data were created or analysed in this narrative review. All sources were cited in the article.

## Appendix A

**Practical Precautions Summary.** Advice for HFNO use during high-fire-risk procedures.
